# A SAGE-based screen for genes expressed in sub-populations of neurons in the mouse dorsal root ganglion

**DOI:** 10.1186/1471-2202-8-97

**Published:** 2007-11-19

**Authors:** Steeve Bourane, Ilana Méchaly, Stéphanie Venteo, Alain Garces, Agnes Fichard, Jean Valmier, Patrick Carroll

**Affiliations:** 1 INSERM U.583, 34091 Montpellier, France; 2 Université Montpellier II. 34095 Montpellier, France

## Abstract

**Background:**

The different sensory modalities temperature, pain, touch and muscle proprioception are carried by somatosensory neurons of the dorsal root ganglia. Study of this system is hampered by the lack of molecular markers for many of these neuronal sub-types. In order to detect genes expressed in sub-populations of somatosensory neurons, gene profiling was carried out on wild-type and TrkA mutant neonatal dorsal root ganglia (DRG) using SAGE (serial analysis of gene expression) methodology. Thermo-nociceptors constitute up to 80 % of the neurons in the DRG. In TrkA mutant DRGs, the nociceptor sub-class of sensory neurons is lost due to absence of nerve growth factor survival signaling through its receptor TrkA. Thus, comparison of wild-type and TrkA mutants allows the identification of transcripts preferentially expressed in the nociceptor or mechano-proprioceptor subclasses, respectively.

**Results:**

Our comparison revealed 240 genes differentially expressed between the two tissues (P < 0.01). Some of these genes, CGRP, Scn10a are known markers of sensory neuron sub-types. Several potential markers of sub-populations, Dok4, Crip2 and Grik1/GluR5 were further analyzed by quantitative RT-PCR and double labeling with TrkA,-B,-C, c-ret, parvalbumin and isolectin B4, known markers of DRG neuron sub-types. Expression of Grik1/GluR5 was restricted to the isolectin B4+ nociceptive population, while Dok4 and Crip2 had broader expression profiles. Crip2 expression was however excluded from the proprioceptor sub-population.

**Conclusion:**

We have identified and characterized the detailed expression patterns of three genes in the developing DRG, placing them in the context of the known major neuronal sub-types defined by molecular markers. Further analysis of differentially expressed genes in this tissue promises to extend our knowledge of the molecular diversity of different cell types and forms the basis for understanding their particular functional specificities.

## Background

The primary receptor cells of the somatosensory system are the neurons of the dorsal root ganglia (DRG). Their role is to detect environmental stimuli such as noxious stimuli, temperature, mechanical pressure, proprioception and to conduct these signals to the central nervous system. Noxious stimuli are sensed by nociceptors that innervate the skin, muscle and internal organs. Touch and proprioceptive stimuli are detected by low-threshold mechanoreceptors that innervate the skin and muscle, respectively.

There exist up to 20 different sub-types of sensory neurons in the DRG [1]. Different sub-types can be distinguished by a variety of criteria: cell body diameter; conduction velocity; neurotrophic factor dependence; sensitivity to specific stimuli; expression of neuropeptides, ion channels, calcium-binding proteins and transcription factors [2]. However, for many sub-types no specific molecular markers have been found. For example, slowly adapting mechanoreceptors [SAMs] that interact with Merkel cells in the skin and whose phenotypic development depends on BDNF/TrkB signaling [3] can only be identified using physiological criteria. Thus identification of specific molecular markers is an essential step in understanding the development and the function of this system. A large body of evidence shows that nociceptors depend for their survival during development on NGF signaling through TrkA receptors specifically expressed on these cells [4-6]. TrkA mutant mice are insensitive to painful stimuli and mutations in TRKA are associated with the syndrome "Congenital insensitivity to pain" in humans [7]. Around the time of birth, nociceptors divide into 2 main populations, one of which maintains TrkA expression and secretes neuropeptides such as CGRP and substance P. The other population down-regulates TrkA, expresses the Ret tyrosine kinase receptor and requires GDNF for its survival [8]. This non-peptidergic population is further characterized by the capacity of binding the lectin IB4 and it has recently been shown that the transcription factor Runx1 is necessary for the phenotypic development of this cell population [9]. In the adult mouse, peptidergic (TrkA expressing) and non-peptidergic (c-Ret expressing) nociceptors project to the different laminae in the dorsal horn, and may be responsible for different pain modalities (reviewed in [10]). Skin mechanoreceptors and muscle proprioceptors depend for their survival on NT-3, BDNF and NT-4 and project to deeper laminae in the spinal cord, reviewed in [2].

To study the physiology of somatosensory neurons and the molecular changes in functionally-identified DRG neuron sub-types during development and after peripheral trauma, we have created several SAGE (serial analysis of gene expression) libraries from DRG tissues [11]. SAGE generates global gene expression data from thousands of transcripts in a given tissue or cell-type [12]. Since nociceptors constitute up to 80% of all neurons in the DRG, transcripts representing this cell type should be enriched in wild-type tissue. TrkA mutant mice lose all nociceptive neurons during development due to inactivation of the NGF survival signaling pathway [4,6], leaving only TrkB and TrkC mechanoreceptor neurons, thus the TrkA mutant DRG is enriched for transcripts representing low-threshold myelinated mechanoreceptors. In the study presented here, we compared the transcription profiles of wild-type and TrkA mutant DRG from neonatal mice in order to identify markers of sub-populations. Double labeling analysis of a selection of these genes with known markers of DRG neuron sub-types revealed expression in sub-populations of DRG neurons in the adult mouse from birth to adulthood.

## Results

### General results from SAGE libraries analysis

**Figure 1:**
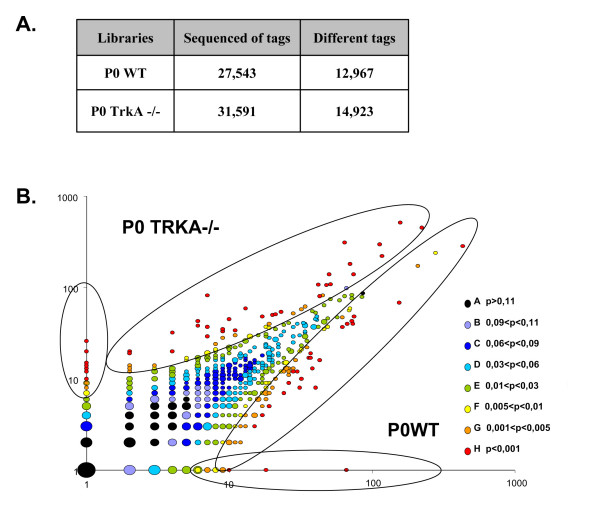
(A) Numbers of sequenced tags and different tags for each library. (B) Scatter plot showing a comparative expression level of tags between P0 WT and TrkA mutant. The occurrence of each tag was plotted on a logarithmic scale (tags with no expression in one library were set to a value of one). A color is attributed to the tag according to the significance of the *P *value. Differently expressed tags (*P < 0.01*) are encircled.

We used SAGE technology to generate global gene expression profiles from wild-type and TrkA mutant DRGs. This methodology consists in isolating, from a given cDNA preparation, short 14-bp tags which are small nucleotides sequences representative of a particular transcript [12]. Sequencing and counting of tags generates information about the presence and frequency of thousands of transcripts in the original tissue. One thousand plasmid inserts were sequenced for each library, resulting in 27,543 and 31,591 tags for the wild-type and the mutant DRG libraries, respectively. The overall results of the bioinformatics analysis are shown in Figure 1.

### Differential analysis of P0 WT versus P0 TrkA mutant mice libraries

Tag frequencies were compared between the WT and TrkA mutant SAGE libraries in order to identify genes preferentially expressed in one or the other tissues. This analysis revealed that 240 genes are differentially expressed (P < 0.01) between the two banks including 151 tags more highly expressed in wild type library and 89 tags more represented in mutant mice library, and might represent new markers of subpopulations of DRG neurons. A preliminary analysis of 20 genes, chosen on the basis of differential Tag numbers, was carried out by whole-mount in situ hybridisation on P0 dorsal root ganglia (not shown). Subsequently we analyzed, by in situ hybridisation on cryostat sections, three genes chosen on the basis of restricted expression pattern and published information about their potential function. Crip2 (Cysteine-rich intestinal protein 2, [GenBank:NM_024223] is an intracellular adaptor protein that may interact with actin cytoskeleton [13] and has never been described in the DRG. Dok4 (Downstream of tyrosine kinase/Docking proteins, [GenBank:NM_053246] has been reported in embryonic DRG neurons without specifying what sub-population and is thought to play a role in receptor tyrosine kinase signalling pathways [14]. Grik1 alias GluR5 (Glutamate receptor, ionotropic, kainate 1, [GenBank:NM_146072] is a glutamate receptor that has been associated with P2X3-expressing non-peptidergic nociceptors in the DRG [15]. Pharmacological studies [16] and analysis of Grik1/GluR5 knockout mice [17] show a role for this molecule in nociception. The SAGE results for these 3 genes as well as for several markers of sensory neuron populations are shown in the Supplementary Table [18]. These genes were further studied in detail by quantitative RT-PCR and in situ hybridisation.

### Quantitative PCR on selected genes

**Figure 2:**
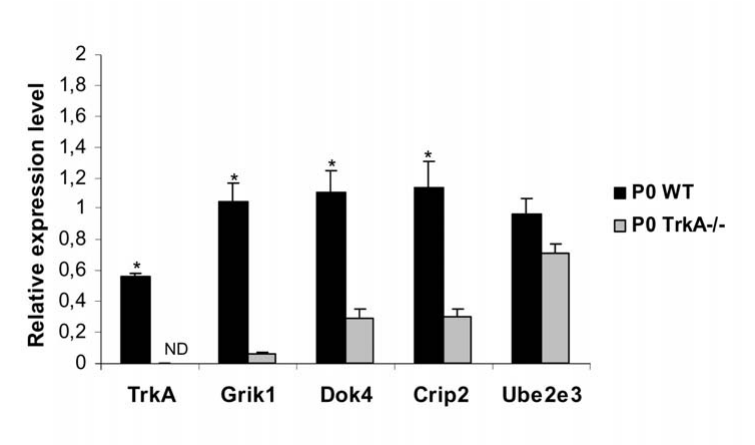
**QRT-PCR**: Gene expression determined by real-time PCR on P0 and P0 TrkA mutant mouse lumbar DRG. TrkA and Ube2e3 (ubiquitin-conjugating enzyme E2E 3) were used as controls. Data (means ± SEM) were calculated by the delta-CT method [37] on three independent experimental replicates. The arithmetic means of the expression levels of two genes (Polr2j, Ddx48) whose expression do not change in the course of development and in TrkA-/- DRG were used to normalize the expression levels. Data were analyzed using the Mann Whitney U-test (*P < 0.05). ND: Not detected.

For the 3 chosen genes we carried out quantitative RT-PCR on mRNA extracted from wild-type and TrkA mutant DRG tissue dissected at P0. Grik1/GluR5, Dok4 and Crip2 transcripts were less abundant in TrkA mutant DRG compared to wild-type; relative normalized expression levels were respectively 1.04 +/- 0.12; 1.10 +/- 0.13; 1.13 +/- 0.16 in wild-type and 0.06 +/- 0.01; 0.29 +/- 0.05; 0.30 +/- 0.04 in Trk A mutant mice (Figure 2). These quantitative RT-PCR results confirmed the data obtained from the SAGE tag analysis. When expressed as "fold-change in expression" the SAGE results demonstrate a down regulation of Grik1, Dock4 and Crip2 expression between P0 WT and P0 Trka-/- DRG of about 18, 6 and 3 fold respectively, while QRT-PCR provided an expression ratio between P0 WT and P0 Trka-/- DRG of 17, 4 and 4 fold respectively.

### In situ hybridization patterns of selected genes

**Figure 3:**
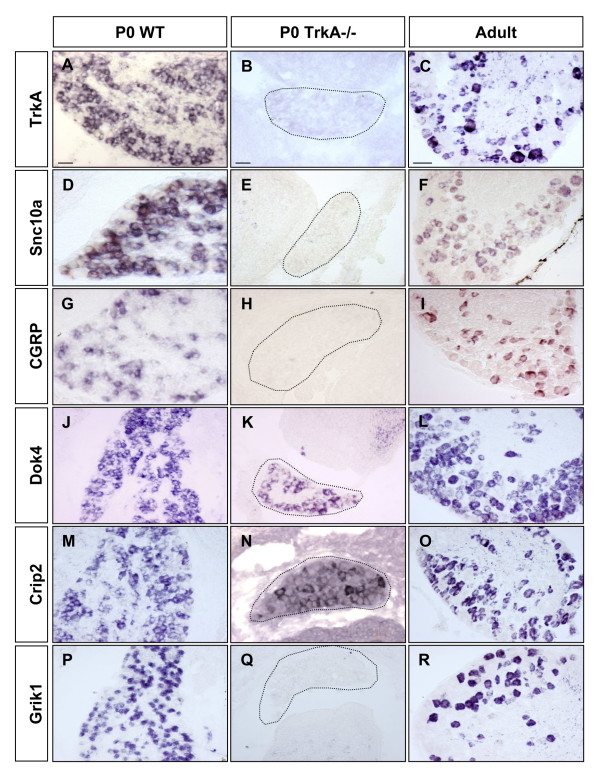
**In situ hybridization patterns of selected genes**: In situ hybridization on cryostat section of P0 Wild type (A, D, G, J, M, P), P0 TrkA mutant (B, E, H, K, N, Q) and adult (C, F, I, L, O, R) with DIG-labelled probes for TrkA (A, B, C); Scn10a (D, E, F); CGRP (G, H, I); Dok4 (J, K, L); Crip2 (M, N, O) and Grik1/GluR5 (P, Q, R). Arrows in L and O indicate unlabelled cells. TrkA mutant DRGs are encircled in B, E, H, K, N and Q. Data show that Dok4 was expressed in the majority of neurons, scattered negative cells was observed, arrows in L. Crip2 was widely expressed, although substantial numbers of negative cells were noted, arrows in O. Grik1/GluR5 was expressed in sub-population of neurons and was completely absent from TrkA mutant DRG. Scale bar 50 μm.

In order to assess the types of cells in which the candidate genes were expressed and to gain idea about the potential expression in functional sub-types, we carried out in situ hybridization on sections of wild-type or mutant ganglia using DIG-labelled probes generated from PCR products amplified using primers specific for each gene. In all cases the PCR products were sequenced to confirm the identity of the probe. Figure 3 shows the in situ hybridization profile for these transcripts on cryostat sections from wild-type P0, TrkA mutant and adult DRGs. As controls we used riboprobes generated against TrkA, the neuropeptide CGRP and the sodium channel Scn10a, all known markers of nociceptive neurons. As shown in Figure 3(A–I) , TrkA, CGRP and Scn10a all label sub-populations of neurons in P0 wild-type and adult DRGs. In accordance with the loss of nociceptive neurons in TrkA mutant DRGs, no labelling for these transcripts was observed in TrkA mutant DRGs (Figure 3B, E, H).

Transcripts representing the adaptor protein Dok4 were observed in broad range of neuron-like cells in wild-type P0 and adult DRG (95% +/- 0.9 of all neurons), with negative cells scattered throughout the tissue (arrows in Figure 3L). Labelled cells were also observed in TrkA mutant DRG sections from new-born mice (Figure 3K).

Crip2 was expressed in a large proportion (86% +/- 1) of cells of neuronal morphology in the P0 wild-type and adult DRG (Figure 3M, O). However, unlabeled cells were observed. In P0 TrkA mutant DRG, a number of Crip2 labelled cells were observed (Figure 3N), suggesting that the expression of this gene is not exclusive to the nociceptor population. Crip2 expression persisted in adult DRG, and negative cells of large cell diameter were observed (Figure 3O, arrows).

The kainate receptor Grik1/GluR5 was expressed in a sub-population of cells of neuronal morphology in wild-type DRG and was completely absent in TrkA mutant DRG, strongly suggesting nociceptor specific expression (Figure 3P, Q, R). Grik1/GluR5 expression persisted in a sub-population of cells (37% +/- 1.3 of all neurons) in the adult (Figure 3R).

### Sub-population specific expression pattern revealed by double labeling

**Figure 4:**
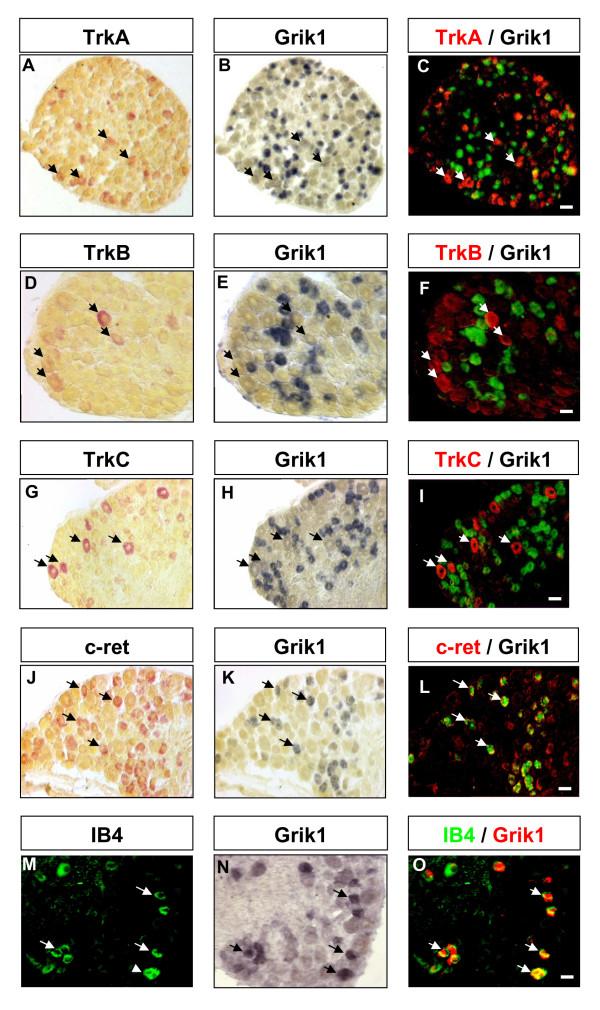
**Co-localisation Grik1/GluR5 mRNA with markers of defined sub-populations of sensory neurons**: Double in situ hybridization was carried out on sections of adult DRG by using fluorescein/fast red detection for TrkA (A), TrkB (D), TrkC (G), c-ret (J) and immunohistochemical detection of isolectin B4 (M). Grik1/GluR5 mRNA was subsequently detected using DIG/NBT-BCIP detection in B, E, H, K, N. In situ signals were converted into pseudo colors and images were superimposed (C, F, I, L, O) to show co-labelling of cells. Grik1/GluR5 was excluded from TrkA, B and C population but was co-localised with c-ret and isolectin B4 neurons. Scale bar 50 μm.

To further characterise the sub-population in which the genes of interest were expressed, we carried out double labelling using known markers of neuronal DRG sub-populations (Figure 4).

Grik1/GluR5 expression was absent from each of the TrkA, TrkB and TrkC expressing populations (Figure 4C, F, I). However, Grik1/GluR5 was expressed in a sub-population (73 % +/- 0.8) of neurons of small cell body diameter labelled by c-Ret (Figure 4L). These cells correspond to the isolectin B4 positive nociceptor population as shown by double labelling where there was 87 % +/- 0.3 co-localisation of Grik1/GluR5 and isolectin B4 (Figure 4O). Virtually all Grik1/GluR5-expressing neurons were IB4-positive.

**Figure 5:**
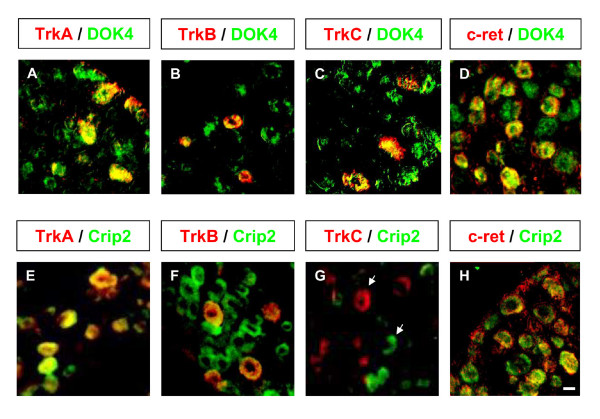
**Co-localisation of Crip2 and Dok4 mRNAs with defined sub-populations of sensory neurons**: Double in situ hybridization was carried out by using fluorescein/fast red detection for TrkA (A, E), TrkB (B, F), TrkC (C, G), c-ret (D, H) and DIG/NBT-BCIP for Dok4 (A, B, C, D) and Crip2 (E, F, G, H). In situ signals were converted into pseudo colors and images were superimposed to show co-labelling of cells. Dok4 co-localised with all major subtypes of DRG neurons, Crip2 was specifically excluded from TrkC population. Scale bar 50 μm.

Dok4 was expressed in a wide range (95 % +/- 0.9) of neurons of different cell diameters in the adult DRG. In co-localisation studies we observed that most neurons expressing members of the Trk receptor family also expressed Dok4. In addition, most c-ret and isolectin B4+ neurons also expressed Dok4 (Figure 5A–D). However, we consistently observed that about 5% of all cells with neuron-like morphology were negative for Dok4. In co-labeling experiments with Trks, c-ret and IB4 lectin, it was observed that these Dok4 negative cells were never labelled by any of the 5 markers used.

Most TrkA-expressing neurons were also Crip2-positive, as were TrkB and c-ret expressing neurons (Figure 5E–H). However, all TrkC expressing neurons were negative for Crip2 (Figure 5G), and as expected, Crip2 was never co-localised with parvalbumin, a definitive marker of muscle proprioceptors (data not shown).

## Discussion

In this report we describe the use of a combination of SAGE analysis and in situ hybridization to identify markers of sensory neuron sub-types in the dorsal root ganglion of the mouse. We took advantage of the fact that in the TrkA mutant mouse all neurons of the nociceptive sub-type die during development. By creating SAGE banks from wild-type (> 75% nociceptive) and TrkA mutant (100% mechanosensitive and proprioceptive) we could compare global expression profiles of the 2 major classes of neurons in the DRG i.e. mainly non-myelinated or thinly-myelinated nociceptors and myelinated low-threshold mechanoreceptors of skin and muscle. The choice of the developmental time-point P0 was determined by the fact that TrkA mutant mice die in the days following birth, and that we are interested in identifying molecules involved in the functioning of DRG neurons in the maturing somatosensory system. Although the starting material, whole DRG, necessarily contains non-neuronal cells including satellite glia and immature Schwann cells, methods based on isolating purified neurons in culture were considered to be overly artificial, since the cultured neurons undergo axotomy during dissection.

We carried out more detailed analysis of a limited sample of these genes, whose exact sub-population specificity had not previously been determined, by QRT-PCR and double labeling using known markers of sensory neuron sub-populations. In each case, the SAGE results were confirmed by quantitative RT-PCR.

Qualitatively, we observed three types of in situ hybridization expression patterns. For Dok4 there is a quantitative difference in expression, while the double in situ hybridization pattern does not reveal remarkable sub-population specificity of expression. We can suggest that while Dok4 is expressed in most neurons, expression is generally higher in the nociceptive population. However, it cannot be ruled out that the basal expression level of this gene is changed in the surviving neurons of the TrkA mutant DRG. Downstream of tyrosine kinase/Docking proteins (Dok) comprise a family of intracellular adaptors that modulate signaling pathways mediated by receptor and non-receptor tyrosine kinases. For example Dok7 regulates neuromuscular junction formation by interaction with MuSK [19]. In biochemical studies, it was shown that Dok5 could interact specifically with TrkB and TrkC receptors, but not TrkA, and was involved in neurotrophin induced MAPK activation [20]. Dok4 was shown to regulate GDNF/Ret dependent neurite outgrowth in neuroblastoma cells [21]. Expression of Dok4 has been described in the DRG during embryonic development, and an interaction with c-Ret was demonstrated in a heterologous cell expression system [14]. Our results show that, in adult DRG, Dok4 is expressed in Ret-expressing neurons, but the broad expression of this molecule suggests a potential role in association with other tyrosine kinase receptors. Interestingly, about 5% of adult DRG neurons were Dok-negative and these cells did not express any of the 4 typical DRG neuron tyrosine kinase receptors (trks A, B, C, c-ret), suggesting that there might be an as yet unidentified sub-population that uses a ligand receptor signaling system other than Trks or Ret. This observation is in line with the study of Kashiba et al., [22] who showed, using a cocktail of in situ hybridization probes for the Trks and Ret receptors, that a small proportion (3%) of neurons remained unlabeled.

Our results show for the first time that Crip2 is expressed in DRG neurons. Intriguingly, this molecule is very specifically excluded from the TrkC-expressing population that includes the parvalbumin expressing proprioceptors [23] and certain low-threshold mechanoreceptors [24]. Thus it is possible that this gene is negatively regulated by TrkC signaling. On the other hand, it has been shown that the transcription factor RunX3 plays an essential role in the establishment of the proprioceptive neuron phenotype [25] and the targeting of proprioceptive (TrkC-expressing) afferents to the ventral region of the spinal cord [9], raising the possibility that Crip2 expression is under the control of this transcriptional program. The cysteine-rich LIM-only protein Crip2 is a member of a family of related proteins, Crip1, Crip2 and TLP/Crip3 [13,26,27]. These proteins contain 1 or 2 LIM domains and are thought to act as intracellular adaptors, although the signaling pathway(s) involved have not been identified. The expression of Crip2 has been described in developing and adult mesenchymal tissue and in particular in the developing and adult heart [26]. An interaction between Crip2 and the mouse protein tyrosine phosphatase PTP-BL [13] was demonstrated by 2-hybrid screen, and it was proposed that Crip2/PTP-BL interactions might play a role in the dynamics of the actin cytoskeleton. The functional significance of Crip2 expression in DRG neurons remains to be explored, Concerning Grik1/GluR5, our results show unequivocally that, in the adult mouse DRG, this kainate receptor is expressed in the great majority of isolectin B4 binding neurons and is excluded from other neuronal sub-types. A body of evidence shows that Grik1/Glur5 receptors are expressed in sensory neurons of the DRG and are transported to the spinal cord where they are an essential component of presynaptic kainate receptors thought to play a role in the modulation of pain sensation [28]. Grik1/GluR5 mutant mice display altered behavioral responses to noxious stimuli [17]. Several studies have *indirectly *pointed to a localization of Grik1/GluR5 in the isolectin B4 binding DRG neuron sub-population. By pharmacological experiments on cultured neonatal rat DRG neurons, Grik1/GluR5 was identified in isolectin B4 binding cells [29] and was shown by double immunhistochemistry to be highly-represented in the P2X3 positive nociceptor population, that are mostly isolectin B4 binding [15]. In previous studies, using in situ hybridization, the expression of Grik1/GluR5 was detected in mouse DRG from E12 onward, using a rat probe on mouse tissue [30], and by RT-PCR in E16 rat DRG tissue [29]. In our experiments on mouse tissue, by both QRT-PCR and in situ hybridization, we could not detect this mRNA at 2 time-points prior to birth, E13 and E15 (data not shown). This discrepancy may be due to the use of cross-species probes in the earlier study, or the fact that earlier in situ studies used more sensitive radioactive in situ technique. Nevertheless, our in situ hybridization and QRT-PCR results suggest that *high *Grik1/GluR5 expression appears at the late embryonic phase. At P0, Grik1/GluR5 was largely co-localized with c-ret (data not shown). In the adult, Grik1/GluR5 never co-localized with TrkA and was restricted to small cell-diameter c-Ret+/isolectin B4+ neurons. Thus Grik1/GluR5 expression seems to coincide with the appearance of the population of nociceptors that down-regulate TrkA and express c-Ret and bind isolectin B4 [8]. This switch in expression also parallels that of Runx1, a transcription factor that is broadly co-localized with TrkA at P0, and whose expression becomes restricted to the isolectin B4+ nociceptor population during early postnatal life [31]. Indeed, Runx1 appears to regulate the molecular phenotype of this cell-type as demonstrated by the analysis of Runx1 mutant mice, in which was observed an expansion of the TrkA/peptidergic nociceptor phenotype and a lack of expression of a range of genes normally expressed in the non-peptidergic/IB4+ nociceptor population [31]. Runx1 mutant mice display loss of sensitivity to acute thermal, but not mechanical, pain stimuli, as well as reduced responses to inflammatory stimuli [31] a phenotype partially mirrored by that of the Grik1/GluR5 mutant [17]. Our results suggest that Grik1/GluR5 could be a target gene of Runx1 and should play a role in the specific functions of these neurons.

## Conclusion

In conclusion, we have identified and characterized the detailed expression patterns of three genes in the developing DRG, placing them in the context of the known major neuronal sub-types as defined by several molecular markers. Further analysis of differentially expressed genes in this tissue promises to extend our knowledge of the molecular diversity of different cell types and forms the basis for understanding their particular functional specificities.

## Methods

### Animals

Procedures involving animals and their care were conducted in agreement with the French Ministry of Agriculture and the European Community Council Directive no. 86/609/EEC, OJL 358, 18 December 1986. Early post-natal (P0) mice, were killed by decapitation. Adult mice were deeply anaesthetized with CO2 and then decapitated. Lumbar L3-L6 DRG for P0 and L4-L5 for adult stages were acutely dissected in ice cold Phosphate buffered saline (PBS). TrkA null mutant mice (Smeyne et al., 1994) were generated by breeding heterozygotes (C57Bl6 background).

### SAGE Library construction and data analysis

SAGE libraries were made on lumbar wild-type and TrkA mutant mice DRGs (approximately 80–100,000 cells) using the I-SAGE™ Kit (Invitrogen, France) according to the manufacturer's protocol and as described previously [11]. Mutant mice were genotyped by PCR before DRG samples are pooled for RNA isolation. Briefly, tissues were transferred in 100 μl of Lysis binding buffer and carefully crushed in a 1 ml dounce homogeniser. Double strand cDNA was synthesised from mRNA using oligo (dT)25 as primer for the first strand synthesis and was digested with the sequence specific anchoring restriction enzyme Nla III. cDNA was divided in two fractions and ligated with two adapters A and B containing cohesive 4 bp overhangs complementary to the Nla III digested cDNA, a tagging enzyme BsmfI recognition site at the 3' end and priming sites for PCR amplification. Subsequent digestion with BsmfI released adapters with an anchored short piece of cDNA (10–14 bp) corresponding to a unique sequence from a single transcript. After blunt-ending of tags the two fractions of tags were ligated to form 110 bp ditags. Ditags were then amplified in a large scale PCR and 26 bp ditags were recovered after Nla III digestion, and polyacrylamide gel purification. Ditags were then concatenated by self ligation, gel purified and then cloned into pZERO-1 vector to obtain a SAGE library. About 1000 randomly selected clones were sequenced for each library (Genome Express, France). The number of tags per clone was between 30 and 45. Several improvements of the classical protocol were made [32-34].

The analysis of the generated data, and in particular, sequence data analysis assessing the quality of the library, extraction of tag sequences from concatemers, their annotations and the analysis of their distributions was carried out using bioinformatic tools developed by the company Skuld-tech [18] as previously described [11]. Calculation of P-values for the statistical significance of the differences in tag frequencies for given genes between two SAGE libraries was performed as described previously [35].

### Quantitative RT-PCR

Quantitative RT-PCR was conducted as previously described [11]. Briefly, total RNA was extracted from P0 wild-type and Trka mutant mice lumbar DRG in TriReagent solution (Sigma), quantified by optical densitometry and its integrity was checked by electrophoresis on agarose gels. Total RNA was DNase treated with RQ1 DNase (Promega) according to manufacturer's instructions. 1 μg of total RNA was reverse-transcribed 1 hour at 37°C with 100 U of Superscript II reverse transcriptase (Invitrogen) and 5 μM hexamer random primers (Roche), 0,5 mM of each dNTPs (Invitrogen), 10 mM of dithiothreitol and 20 U of recombinant RNase inhibitor (Promega). Real time PCR was performed by using SYBR Green I dye detection on the LightCycler system (Roche Molecular Biochemichals). PCR reactions were carried out in a 10 μl volume containing 3 μl of RT product (final dilution 1/30), 0,5 μM of forward and reverse primers, and 5 μl of QuantiTect SYBR Green PCR Master Mix (Qiagen). After an initial activation step of 15 min at 95°C, 45 cycles consisting of 94°C for 15s, 55°C for 20 sec and 72°C for 35 sec were performed. The identity of RT-PCR amplified products was confirmed by sequencing (Genome express, France) and routinely by finishing PCR runs with a melting curve analysis (70°C to 95°C at 0,2°C increments).

The following primer pairs were used to generate the PCR products

TrkA (primers detect all of the 4 known TrkA transcript variants; [GenBank:XM_986774, XM_986810, XM_986852, XM_986890]:

s- TGGCAGTTCTCTTTCCCCTA; as- AAAGCTCCACACATCGCTCT

Grik1/GluR5 [GenBank:NM_146072]:

s- CACATTCAGACTCGCTGGAA; as- CTCCTTGAGCAGAGGTTTGG

Dok4 [GenBank:NM_053246]:

s- CACTGTCCGTGGAATGTCTG; as- GGTAAAGCGCGTAGCATCTC

Crip2 [GenBank:NM_024223]:

s- AGGCACAGTTCAGCCCTAGA; as- GGACAGTCACCTTGGAGAGC

Ube2e3 [GenBank:NM_009454]:

s- TGCTGGGCCTAAAGGAGATA; as- TCCCTGACTGTTGATGTTGC

Polr2j [GenBank:NM_011293]:

s- ACCACACTCTGGGGAACATC; as- CTCGCTGATGAGGTCTGTGA

Ddx48 [GenBank:NM_138669]:

s- GGAGTTAGCGGTGCAGATTC; as- AGCATCTTGATAGCCCGTGT

The relative amounts of specifically amplified cDNAs were calculated using the delta-CT method [36,37] on three independent experimental replicates after normalisation by two stable control genes (polymerase (RNA) II polypeptide J, polr2j and DEAD box polypeptide 48, Ddx48). The Mann Whitney U-test was used for comparison between groups. p value < 0,05 were considered statistically significant.

### Amplification of selected genes by RT-PCR and DIG-labeled riboprobe synthesis

PCR products of 400–800 bps were amplified from wild-type mouse cortex or DRG cDNA as template, using primers designed using the Primer 3 software [38], in a 20 μl reaction using Platinum Taq polymerase (Invitrogen) under standard conditions. All PCR products used in subsequent experiments were sequenced (Millegene, France). The following primer pairs were used to generate the PCR products of about 500 base-pairs:

TrkA (primers detect all of the 4 known TrkA transcript variants; [GenBank:XM_986774, XM_986810, XM_986852, XM_986890]:

s-TGGCAGTTCTCTTTCCCCT; as-AAAGCTCCACACATCGCTCT

Scn10a [GenBank:NM_009134]:

s-GAACCTGACAAAGCCAGCTC; as-AATGCGGTAACAGGTTTTGC

CGRP [GenBank:NM_001033954]:

s-TGGTTGTCAGCATCTTGCTC; as-CAACACGATGCACAATAGGC

Grik1/GluR5 [GenBank:NM_146072]:

s-GAATGACAAAGGGGAGTGGA; as-AAGGTCATTGTCGAGCCA

Crip2 [GenBank:NM_024223]:

s-CCTCCAAGTGTCCCAAGTGT; as-CTGAGGCAGGACTAGGCAAC

Dok4 [GenBank:NM_053246]:

s-TCGTCAAGCAAGGCTATGTG; as-CTGTGAACCCGCTGGTAAAT

PCR products were ligated into the pGEM-T easy vector using the TA cloning kit (Promega) and manufacturer's instructions. In order to generate PCR products carrying the T7 promoter in an appropriate orientation to generate antisense DIG riboprobes, a second PCR amplification was carried out on the above ligation mix using the sense primer for the gene of interest and the M13forward primer located downstream of the T7 promoter sequence in the pGEM-T easy vector. The resulting PCR product contains the T7 RNA polymerase promoter sequence downstream of the cDNA fragment. Antisense DIG probes were generated in a 5 μl reaction containing 100–200 ng PCR products and using DIG RNA labeling mix (Roche) and T7 RNA polymerase (Invitrogen) following the manufacturer's instructions. DIG-labeled riboprobe was ethanol-precipitated with LiCL, washed with 70% ethanol and resuspended in water.

### In situ hybridisation on cryostat sections of DRG

L3-L6 DRG from P0 wild-type and TrkA mutant mice and adult mice were dissected out separately in PBS and fixed in 2% paraformaldehyde (PFA) for 1 h at RT and cryopreserved in 25% sucrose overnight at 4°C before embedding in OCT compound (Tissue-Tek). Sections of 12 μm were cut on a cryostat, collected on ProbeOn Plus microscope slides (Fisher Scientific) and store at -80°C until used.

In situ hybridizations were performed essentially as described in [39]. After hybridization overnight at 65°C with a riboprobe, the slices were washed twice in 1× SSC, 50% formamide, and 0.1% Tween-20 at 65°C for 30 min and blocked in the presence of 2% blocking reagent and 20% inactivated sheep serum. The slides were then incubated with anti-DIG-alkaline-phosphatase (AP)-conjugated antibody (Roche Diagnostics), washed and revealed with NBT/BCIP staining.

### Double in situ hybridization/immunhistochemistry

Double in situ hybridizations were performed on 12 μm thick frozen sections prepared as above. DIG- and Fluo-labeled probes were mixed in hybridization buffer and applied to sections. After hybridization at 65°C overnight, sections were washed twice in 50% Formamide, 1× SSC, 0.1% Tween-20 for 1 h at 65°C, twice in MABT buffer for 30 min before blocking in blocking buffer (MABT, 2% blocking reagent from Roche, 20% inactivared sheep serum) for 2 h at room temperature. Sections were then exposed to a 1:2000 dilution of anti-Fluo-alkaline phosphatase (AP)-conjugate antibody (Roche) in blocking buffer overnight at 4°C. After washing for 30 min in MABT, the bound Fluo-probe was visualized by an AP-catalyzed color reaction using Fast Red tablets (Roche) according to the manufacturer's instructions. The color reaction was stopped in water, the slides were mounted temporarily in 90% glycerol and 0.1 M Tris, pH 8.2 and the images were acquired on a Zeiss Axioskop using Samba software. After washing for 15 min in 0.1 M Tris pH 8.2, the AP activity was then inactivated by incubating with 100 mM glycine and 0.1% Tween-20, pH 2.2, for 30 min. The sections were washed twice in PBS, 0.1% Tween-20 for 15 min, post-fixed in 4% PFA in PBS for 10 min at room temperature, washed in PBS, 0.1% Tween-20 for 20 min, twice in MABT buffer for 30 min, blocked again in blocking buffer for 2 h, and incubated overnight with anti-DIG-AP conjugate antibody (Roche) at 4°C. After washing as above, slides were incubated this time with NBT-BCIP (Roche) staining solution according to the manufacturer's instructions and the reaction stopped by washing in water. Fast Red precipitates were then removed by incubating the slides in increasing concentrations of ethanol, culminating in two final incubations in 100% ethanol for 10 min. Photographs of the NBT/BCIP results were then taken for comparison with those showing the Fast Red results on the same sections. The Fast Red signals were converted into pseudo-red and the NBT/BCIP signals into pseudo-green fluorescent colour. The pseudo-red fluorescent images were then carefully overlaid with pseudo-green fluorescent images. This sequential approach permits unequivocal identification of coexpression at the single cell level.

For double staining with isolectin B4, in situ hybridization with digoxigenin (DIG)-labeled probe was first performed. The cryostat sections were blocked in 1% BSA plus 0.1% Triton in PBS for 1 h, followed by staining with IB4-Biotin (10 mg/ml, Sigma) and ExtrAvidin-FITC conjugated (Sigma). After acquisition of fluorescent images, the sections were blocked with 20% sheep serum, incubated with alkaline phosphatase (AP)-conjugated sheep anti-DIG antibody (Roche), followed by AP reaction with NBT/BCIP substrate to label mRNA. The in situ hybridisation signals were then photographed under transluminescent light and converted into pseudo-red fluorescent colour. The IB4+ fluorescent images (green) and in situ images (red) were overlaid to reveal co-labelled cells.

## Cell counting

To determine the percentages of neurons expressing molecular markers, only cells with neuronal morphology and clearly identifiable nuclei were counted manually. A minimum of 4 sections from DRGs were counted and the average numbers of labelled neurons over total numbers of neurons was calculated.

## Authors' contributions

SB carried out in situ hybridization and real time PCR and realised the statistical analysis.

IM generated the SAGE libraries and participated in the bioinformatical analysis of tags and in the drafting of the manuscript.

SV set up and carried out the double in situ hybridization and contributed to the drafting of the manuscript.

AG contributed to the bioinformatical analysis and sequences validation of SAGE tags and in the drafting of the manuscript.

AF contributed to the bioinformatical analysis and sequences validation of SAGE tags.

JV contributed to the conceptual development of the project.

PC is the principal investigator on this project and wrote the final draft of the manuscript. All authors read and approved the final manuscript.

## References

[B1] Petruska JC, Napaporn J, Johnson RD, Gu JG, Cooper BY (2000). Subclassified acutely dissociated cells of rat DRG: histochemistry and patterns of capsaicin-, proton-, and ATP-activated currents. J Neurophysiol.

[B2] Marmigere F, Ernfors P (2007). Specification and connectivity of neuronal subtypes in the sensory lineage. Nat Rev Neurosci.

[B3] Carroll P, Lewin GR, Koltzenburg M, Toyka KV, Thoenen H (1998). A role for BDNF in mechanosensation. Nat Neurosci.

[B4] Smeyne RJ, Klein R, Schnapp A, Long LK, Bryant S, Lewin A, Lira SA, Barbacid M (1994). Severe sensory and sympathetic neuropathies in mice carrying a disrupted Trk/NGF receptor gene. Nature.

[B5] Liebl DJ, Klesse LJ, Tessarollo L, Wohlman T, Parada LF (2000). Loss of brain-derived neurotrophic factor-dependent neural crest-derived sensory neurons in neurotrophin-4 mutant mice. Proc Natl Acad Sci U S A.

[B6] Silos-Santiago I, Molliver DC, Ozaki S, Smeyne RJ, Fagan AM, Barbacid M, Snider WD (1995). Non-TrkA-expressing small DRG neurons are lost in TrkA deficient mice. J Neurosci.

[B7] Indo Y, Tsuruta M, Hayashida Y, Karim MA, Ohta K, Kawano T, Mitsubuchi H, Tonoki H, Awaya Y, Matsuda I (1996). Mutations in the TRKA/NGF receptor gene in patients with congenital insensitivity to pain with anhidrosis. Nat Genet.

[B8] Molliver DC, Wright DE, Leitner ML, Parsadanian AS, Doster K, Wen D, Yan Q, Snider WD (1997). IB4-binding DRG neurons switch from NGF to GDNF dependence in early postnatal life. Neuron.

[B9] Chen AI, de Nooij JC, Jessell TM (2006). Graded activity of transcription factor Runx3 specifies the laminar termination pattern of sensory axons in the developing spinal cord. Neuron.

[B10] Snider WD, McMahon SB (1998). Tackling pain at the source: new ideas about nociceptors. Neuron.

[B11] Mechaly I, Bourane S, Piquemal D, Al-Jumaily M, Venteo S, Puech S, Scamps F, Valmier J, Carroll P (2006). Gene profiling during development and after a peripheral nerve traumatism reveals genes specifically induced by injury in dorsal root ganglia. Mol Cell Neurosci.

[B12] Velculescu VE, Zhang L, Vogelstein B, Kinzler KW (1995). Serial analysis of gene expression. Science.

[B13] van Ham M, Croes H, Schepens J, Fransen J, Wieringa B, Hendriks W (2003). Cloning and characterization of mCRIP2, a mouse LIM-only protein that interacts with PDZ domain IV of PTP-BL. Genes Cells.

[B14] Grimm J, Sachs M, Britsch S, Di Cesare S, Schwarz-Romond T, Alitalo K, Birchmeier W (2001). Novel p62dok family members, dok-4 and dok-5, are substrates of the c-Ret receptor tyrosine kinase and mediate neuronal differentiation. J Cell Biol.

[B15] Lucifora S, Willcockson HH, Lu CR, Darstein M, Phend KD, Valtschanoff JG, Rustioni A (2006). Presynaptic low- and high-affinity kainate receptors in nociceptive spinal afferents. Pain.

[B16] Kerchner GA, Wilding TJ, Li P, Zhuo M, Huettner JE (2001). Presynaptic kainate receptors regulate spinal sensory transmission. J Neurosci.

[B17] Ko S, Zhao MG, Toyoda H, Qiu CS, Zhuo M (2005). Altered behavioral responses to noxious stimuli and fear in glutamate receptor 5 (GluR5)- or GluR6-deficient mice. J Neurosci.

[B18] http://www.skuldtech.com/Msage.htm.

[B19] Okada K, Inoue A, Okada M, Murata Y, Kakuta S, Jigami T, Kubo S, Shiraishi H, Eguchi K, Motomura M, Akiyama T, Iwakura Y, Higuchi O, Yamanashi Y (2006). The muscle protein Dok-7 is essential for neuromuscular synaptogenesis. Science.

[B20] Shi L, Yue J, You Y, Yin B, Gong Y, Xu C, Qiang B, Yuan J, Liu Y, Peng X (2006). Dok5 is substrate of TrkB and TrkC receptors and involved in neurotrophin induced MAPK activation. Cell Signal.

[B21] Uchida M, Enomoto A, Fukuda T, Kurokawa K, Maeda K, Kodama Y, Asai N, Hasegawa T, Shimono Y, Jijiwa M, Ichihara M, Murakumo Y, Takahashi M (2006). Dok-4 regulates GDNF-dependent neurite outgrowth through downstream activation of Rap1 and mitogen-activated protein kinase. J Cell Sci.

[B22] Kashiba H, Uchida Y, Senba E (2003). Distribution and colocalization of NGF and GDNF family ligand receptor mRNAs in dorsal root and nodose ganglion neurons of adult rats. Brain Res Mol Brain Res.

[B23] Klein R, Silos-Santiago I, Smeyne RJ, Lira SA, Brambilla R, Bryant S, Zhang L, Snider WD, Barbacid M (1994). Disruption of the neurotrophin-3 receptor gene trkC eliminates la muscle afferents and results in abnormal movements. Nature.

[B24] Airaksinen MS, Koltzenburg M, Lewin GR, Masu Y, Helbig C, Wolf E, Brem G, Toyka KV, Thoenen H, Meyer M (1996). Specific subtypes of cutaneous mechanoreceptors require neurotrophin-3 following peripheral target innervation. Neuron.

[B25] Kramer I, Sigrist M, de Nooij JC, Taniuchi I, Jessell TM, Arber S (2006). A role for Runx transcription factor signaling in dorsal root ganglion sensory neuron diversification. Neuron.

[B26] Yu TS, Moctezuma-Anaya M, Kubo A, Keller G, Robertson S (2002). The heart LIM protein gene (Hlp), expressed in the developing and adult heart, defines a new tissue-specific LIM-only protein family. Mech Dev.

[B27] Kirchner J, Forbush KA, Bevan MJ (2001). Identification and characterization of thymus LIM protein: targeted disruption reduces thymus cellularity. Mol Cell Biol.

[B28] Kerchner GA, Wilding TJ, Huettner JE, Zhuo M (2002). Kainate receptor subunits underlying presynaptic regulation of transmitter release in the dorsal horn. J Neurosci.

[B29] Lee CJ, Kong H, Manzini MC, Albuquerque C, Chao MV, MacDermott AB (2001). Kainate receptors expressed by a subpopulation of developing nociceptors rapidly switch from high to low Ca2+ permeability. J Neurosci.

[B30] Bettler B, Boulter J, Hermans-Borgmeyer I, O'Shea-Greenfield A, Deneris ES, Moll C, Borgmeyer U, Hollmann M, Heinemann S (1990). Cloning of a novel glutamate receptor subunit, GluR5: expression in the nervous system during development. Neuron.

[B31] Chen CL, Broom DC, Liu Y, de Nooij JC, Li Z, Cen C, Samad OA, Jessell TM, Woolf CJ, Ma Q (2006). Runx1 determines nociceptive sensory neuron phenotype and is required for thermal and neuropathic pain. Neuron.

[B32] Powell J (1998). Enhanced concatemer cloning-a modification to the SAGE (Serial Analysis of Gene Expression) technique. Nucleic Acids Res.

[B33] Virlon B, Cheval L, Buhler JM, Billon E, Doucet A, Elalouf JM (1999). Serial microanalysis of renal transcriptomes. Proc Natl Acad Sci U S A.

[B34] Datson NA, van der Perk-de Jong J, van den Berg MP, de Kloet ER, Vreugdenhil E (1999). MicroSAGE: a modified procedure for serial analysis of gene expression in limited amounts of tissue. Nucleic Acids Res.

[B35] Piquemal D, Commes T, Manchon L, Lejeune M, Ferraz C, Pugnere D, Demaille J, Elalouf JM, Marti J (2002). Transcriptome analysis of monocytic leukemia cell differentiation. Genomics.

[B36] Hoebeeck J, van der Luijt R, Poppe B, De Smet E, Yigit N, Claes K, Zewald R, de Jong GJ, De Paepe A, Speleman F, Vandesompele J (2005). Rapid detection of VHL exon deletions using real-time quantitative PCR. Lab Invest.

[B37] Vandesompele J, De Preter K, Pattyn F, Poppe B, Van Roy N, De Paepe A, Speleman F (2002). Accurate normalization of real-time quantitative RT-PCR data by geometric averaging of multiple internal control genes. Genome Biol.

[B38] Rozen S, Skaletsky H (2000). Primer3 on the WWW for general users and for biologist programmers. Methods Mol Biol.

[B39] Carroll P, Gayet O, Feuillet C, Kallenbach S, de Bovis B, Dudley K, Alonso S (2001). Juxtaposition of CNR protocadherins and reelin expression in the developing spinal cord. Mol Cell Neurosci.

